# Identifying characteristics of adolescents with persistent loneliness during COVID‐19: A multi‐country eight‐wave longitudinal study

**DOI:** 10.1002/jcv2.12206

**Published:** 2023-11-08

**Authors:** Laura Riddleston, Meenakshi Shukla, Iris Lavi, Eloise Saglio, Delia Fuhrmann, Rakesh Pandey, Tushar Singh, Pamela Qualter, Jennifer Y. F. Lau

**Affiliations:** ^1^ Department of Psychology King’s College London Institute of Psychiatry, Psychology & Neuroscience (IoPPN) London UK; ^2^ Department of Psychology University of Allahabad Prayagraj India; ^3^ Department of Psychology University of Bath Bath UK; ^4^ School of Social Work University of Haifa Haifa Israel; ^5^ Department of Psychology Banaras Hindu University Varanasi India; ^6^ Manchester Institute of Education The University of Manchester School of Environment, Education and Development Manchester UK; ^7^ Youth Resilience Unit Centre for Psychiatry and Mental Health Wolfson Institute of Population Health, Queen Mary University of London London UK

**Keywords:** adolescent mental health, COVID‐19 pandemic, perceived social isolation, social restrictions, youth loneliness

## Abstract

**Background:**

Elevated loneliness experiences characterise young people. While loneliness at this developmental juncture may emerge from age‐typical upheaval in social relationships, there is little data on the extent to which young people experience high and persistent levels of loneliness, and importantly, who is most vulnerable to these experiences. Using the widespread social restrictions associated with the COVID‐19 pandemic, which precipitated loneliness in many, we aimed to examine adolescents' loneliness profiles across time and the demographic predictors (age, sex, and country) of more severe trajectories.

**Methods:**

Participants aged 12–18 years, recruited into a multi‐wave study (*N* = 1039) across three sites (UK, Israel, and India) completed a 3‐item loneliness measure fortnightly across 8 timepoints during the pandemic.

**Results:**

Latent class growth analysis suggested 5 distinct trajectories: (1) low stable (33%), (2) low increasing (19%), (3) moderate decreasing (17%), (4) moderate stable (23%), and (5) high increasing (8%). Females and older adolescents were more likely to experience persistently high loneliness.

**Conclusions:**

These findings indicate a need for interventions to reduce loneliness in adolescents as we emerge from the pandemic, particularly for those groups identified as being at highest risk.

## Graphical abstract

We examined adolescents' trajectories of loneliness during a time of social restrictions due to the COVID‐19 pandemic, and whether demographic characteristics (age, sex, and country) predicted more severe loneliness over time. The data indicated five different trajectories of loneliness in adolescents, with a significant proportion experiencing persistently high or increasing loneliness. Females and older adolescents were at higher risk of experiencing severe loneliness.

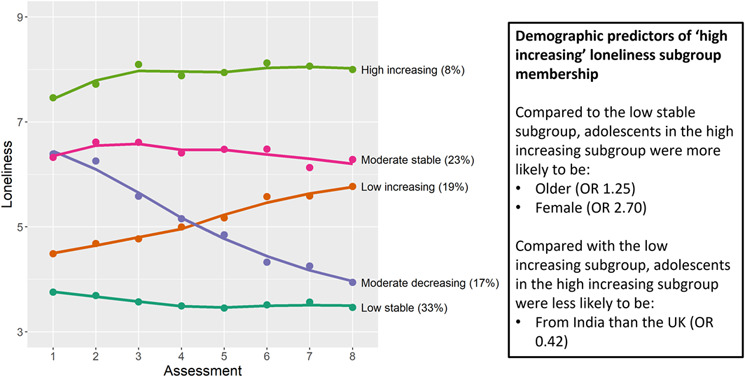

**Key points**

- Loneliness is common among young people and is associated with negative physical and mental health outcomes. However, little is known about the extent to which young people experience high and persistent levels of loneliness, or who is most vulnerable to these experiences.
- We examined adolescents' trajectories of loneliness during a time of social restrictions due to the COVID‐19 pandemic, and whether demographic characteristics (age, sex, and country) predicted more severe loneliness over time.
- The data indicated different trajectories of loneliness in adolescents, with a significant proportion experiencing persistently high or increasing loneliness. Females and older adolescents were at higher risk of experiencing severe loneliness.
- These findings suggest a need for intervention and indicate who might benefit most from this.

## INTRODUCTION

Loneliness, defined as the distressing emotional state that occurs when there is “a discrepancy between one's desired and achieved levels of social relations” (Peplau & Perlman, 1982 In Peplau & Perlman, 1982) is a major public health concern (Leigh‐Hunt et al., 2017). Although often regarded as a problem for older adults, adolescents and young adults also frequently experience loneliness (Barreto et al., 2021; Office for National Statistics, 2018a). Short‐term loneliness in youth could reflect an age‐normative response that encourages re‐connection with others during a period of social transitions (Laursen & Hartl, 2013; Qualter et al., 2015). However, severe forms such as frequent and persistent loneliness at this age are associated with mental (Loades et al., 2020) and physical (van Dulmen & Goossens, 2013) health costs. Yet, little is known about which young people are most vulnerable to these health costs. Here, we explored individual differences in young peoples' trajectories of loneliness at a time of social restriction and potential social isolation (the COVID‐19 pandemic and associated “lockdowns”), and demographic predictors for experiencing persistently high loneliness. We focussed on young people aged between 12 and 18 residing in the UK, Israel, and India, to assess the generalisability of findings.

Pre‐pandemic longitudinal studies of young people (ranging from 7 to 21 years old) have demonstrated individual differences in whether loneliness fluctuates or persists over time, reporting between 2 and 6 subpopulations with different loneliness trajectories within their samples (Benner, 2011; Eccles et al., 2020; Harris et al., 2013; Hutten et al., 2021; Jobe‐Shields et al., 2011; Ladd & Ettekal, 2013; Qualter et al., 2013; Schinka et al., 2013; Vanhalst et al., 2013). Although the largest percentage of young people typically show stable low levels of loneliness across time (van Dulmen & Goossens, 2013), most studies find evidence for groups of young people with unremitting and high levels of loneliness, corresponding to a “persistent” trajectory, making up 3%–22% of the sample (Ladd & Ettekal, 2013; Qualter et al., 2013; Schinka et al., 2013; Vanhalst et al., 2013). Interestingly, some young people show increasing loneliness, starting from low or moderate levels (Benner, 2011; Hutten et al., 2021; Jobe‐Shields et al., 2011; Vanhalst et al., 2013). Those with persistently high or increasing loneliness appear vulnerable to poor mental and physical health, and psychosocial functioning (Hutten et al., 2021; Ladd & Ettekal, 2013; Qualter et al., 2013; Schinka et al., 2013; Vanhalst et al., 2013).

Despite growing evidence for the association between persistently high loneliness and negative health outcomes in young people, research into potential demographic risk factors for persistent loneliness is scarce. General population studies highlight that the propensity to experience loneliness varies across ages even within youth. These studies mark late adolescence and young adulthood as a period of heightened loneliness (Barreto et al., 2021; Victor & Yang, 2012), but less is known about the early‐to‐mid adolescent juncture (i.e. those under 16). Interestingly, Office for National Statistics (ONS) data indicates that more 10–12 year olds in the UK report “often” feeling lonely compared to 13–15 year olds (Office for National Statistics, 2018a). Dynamic social transitions in early to mid‐adolescence, paired with protracted maturation of social‐processing regions of the brain, could make younger adolescents similarly vulnerable to social stressors such as peer rejection and exclusion (Blakemore & Mills, 2014; Wong et al., 2018). Yet, it remains unclear whether persistent loneliness emerges at this juncture. Clarifying this is important for informing when interventions are most needed.

A growing corpus of work has explored male to female differences in youth loneliness, but these studies are mostly cross‐sectional, with mixed results. While some have reported elevated rates of loneliness in females (Moksnes et al., 2022; Yang et al., 2020), another found no differences between males and females (Matthews et al., 2019). A recent meta‐analysis reported slightly higher loneliness in males than females during childhood and adolescence (Maes et al., 2019). Two longitudinal studies in young people did not find differences between males and females' changes in loneliness over time (Schinka et al., 2013; Vanhalst et al., 2013). Thus, when considering persistent rather than transient loneliness, it is also unclear whether being male or female is a risk factor.

Most studies on youth loneliness have been conducted in Western samples, raising questions over whether the presence of more severe forms and whether age and sex confer vulnerability extend to non‐Western cultures. Adult data suggest that culture could shape the experience of loneliness (Rokach et al., 2001; van Staden & Coetzee, 2010), some suggest loneliness may be lower in individualistic than collectivist countries (Dykstra, 2009), while another reports the opposite association (Barreto et al., 2021). In children and adolescents, studies conducted across different countries are even more limited, but where these have been conducted, no differences in mean loneliness were found between 9 and 12‐year‐olds in Brazil, Canada, China, and Italy (Chen et al., 2004), or between Belgian and Chinese adolescents (Maes et al., 2016). We conducted our study across the UK, Israel, and India to diversify studies of youth loneliness beyond Western cultures, providing valuable insight into the generalisability of what constitutes “severe” loneliness.

We aimed to examine changes in adolescents' loneliness over time during the COVID‐19 pandemic and investigate whether there were adolescents who showed more persistent forms of loneliness. We investigated whether demographic characteristics (age, sex, and country) predicted persistently high loneliness. We addressed these questions using multi‐wave loneliness data gathered from 12 to 18‐year‐olds in the UK, Israel, and India. The widespread social restrictions associated with the pandemic represent a universal threat of social isolation, which in turn may predict individual differences in proneness to less adaptive forms of loneliness. There is currently a lack of studies examining loneliness experiences in young people during the pandemic, particularly in those aged 18 or younger. A study of young adults (aged 18–25) in the UK found that loneliness followed a U‐shaped trajectory between June and November 2020 (Hu & Gutman, 2021). In Australian adolescents aged 10–17, feelings of isolation increased during the pandemic, compared with pre‐pandemic levels (Houghton et al., 2022). A longitudinal study of UK adults during the early pandemic identified 4 distinct loneliness trajectories in the sample, one of which reflected a group with persistent high loneliness and made up 14% of the sample (Bu et al., 2020). Younger age and being female were associated with being in this “high” loneliness subgroup (Bu et al., 2020). Based on pre‐pandemic research for this age group (and adult pandemic data), we hypothesised that we would find individual differences in loneliness trajectories in 12‐18‐year‐olds, and that one of these subgroups would show persistently high loneliness. We did not have directional hypotheses for age within adolescence, sex, or country on loneliness trajectory.

## MATERIALS AND METHODS

### Participants and procedure

We analysed data from a multi‐wave study of adolescents across three sites: the UK, Israel, and India. The study was set up to understand and monitor the impact of the COVID‐19 pandemic on young people's emotional wellbeing. Participants completed an initial baseline assessment and seven fortnightly follow‐up survey assessments. Data collection was between May 2020‐April 2021 in the UK, May‐September 2020 in Israel, and June‐October 2020 in India. For further details of assessment data collection dates and length of time between assessments see Tables S1 and S2. The UK study received ethical approval from the Psychiatry, Nursing and Midwifery Research Ethics Committee at Kings College London (Ref: HR‐19/20–18250), the Israel study from the Ethics Committee for Human Experiments, University of Haifa (Ref: 368/20) and from the Psychology Ethics Committee, University of Bath (Ref: 4688 20–05469), and the India study from the Institutional Ethics Committee, Institute of Medical Sciences, Banaras Hindu University, India (Ref: Dean/2020/EC/1975). Inclusion criteria were ability to read the questionnaires presented in the language of that country (English, Hebrew, Hindi), being aged 12–18 in the Israel and India studies and 12–25 in the UK study (to enable cross‐country comparisons only data from UK 12‐18‐year‐olds are presented here), and residing in either the UK, Israel, or India at the time of data collection.

The UK sample was recruited via advertising within schools, colleges and Universities, research advertisement websites, social media, and charities. As this project was funded by a grant to JL, participants were offered a £10 voucher for fully completing four assessments, and another £10 voucher for completing a further four assessments. The survey, information sheets and consent forms were in English. The majority of the Israeli sample were enroled with an Israeli survey company (iPanel), and received compensation for participation (about 5 shekels, an equivalent of 1.5 USD) (through a start‐up fund to IL). The remaining participants were recruited through snowballing. The survey, information sheets and consent forms were in Hebrew. The India sample was recruited by circulating information about the survey through social media. Although all participants were Hindi‐speaking Asian‐Indians, the survey, information sheets and consent forms were available bilingually in Hindi and English and participants could choose their preferred language. Participants aged 16 or over (UK) or 18 years (Israel and India) provided informed consent. For participants under 16 (UK) or 18 years (Israel and India), informed assent and consent was provided by participants and their parent or guardian, respectively. The survey consisted of a battery of measures administered through the online platform, Qualtrics.

Data were cleaned according to each country's protocol, for example, ensuring participant criteria were met, excluding participants with no data, excluding duplicate responses, excluding participants that showed evidence of careless/inauthentic responding (e.g., consistently entering incorrect or nonsensical responses). Participants were included in the current analyses if they had data on the key variable (loneliness) at 5 or more time points. There was no difference in age between participants who were included in the final sample and those who were excluded due to not having sufficient loneliness data (*t*(2131.70) −1.63, *p* = 0.103, *d* = 0.07). However, males were more likely to be excluded than females (*χ* ^2^(1) = 14.99, *p* < 0.001), and participants in the India sample were less likely to be excluded than participants from the Israel or UK samples (*χ* ^2^(2) = 29.16, *p* < 0.001). The final sample for the current analyses consisted of 1039 participants (702 UK; 145 Israel; 192 India). For details of the sample size at each assessment see Table S3.

### Measures

Demographic information: We measured participants' age, sex assigned at birth, and the country they were currently living in.

Loneliness was measured using an adapted version of the 3‐item UCLA loneliness scale (Hughes et al., 2004) recommended for use with children and young people (Office for National Statistics, 2018c) (see Appendix S1 for the full scale). Responses are rated on a 3‐point Likert scale and summed to create a total score. A higher score indicates higher loneliness. Internal consistency at each assessment was good (total sample *α* = 0.79–0.82).

### Statistical analyses

We first explored changes in loneliness over time at the group level using latent growth curve modelling (LGM), performed on data from all three countries using the Lavaan package (version 0.6.8) (Rosseel, 2012) for R (version 4.0.5) (R Core Team, 2021). LGM enables estimation of the initial loneliness level at baseline (intercept) and the change in loneliness over the study duration (slope). The variance in the intercept and slope estimates were examined to identify the presence of individual differences in loneliness trajectories. A linear model was fitted using fixed growth factor loadings of 0, 1, 2, 3, 4, 5, 6, 7, using maximum likelihood estimation with robust Huber‐White standard errors and a scaled test statistic. Good model fit was defined as a comparative fit index (CFI) > 0.97 and Standardised Root Mean Square Residual (SRMR) < 0.05; acceptable fit as CFI = 0.95–0.97, SRMR = 0.05–0.10 (Schermelleh‐Engel et al., 2003).

We then explored individual differences in loneliness trajectories using latent class growth analysis (LCGA), which enables identification of different classes (subgroups) of trajectories (Nagin & Odgers, 2010). LCGA was implemented in MPlus (version 8.4) (Muthén & Muthén, 1998). LCGA is an exploratory method, in which a series of models with an increasing number of latent classes are fitted. We used the following criteria to determine the optimal number of classes for the LCGA model (Ram & Grimm, 2009):

- Model convergence.
- Qualitatively different subgroup trajectories.
- Entropy (measure of subgroup classification quality, acceptable >0.07–0.08).
- Comparing K‐class and K‐1 class model fit statistics, using the Akaike information criterion (AIC), Bayesian information criterion (BIC) and sample size adjusted BIC (aBIC). Lower values indicate better model fit.
- Bootstrapped Lo, Mendell, Rubin likelihood ratio test (LRT), comparing K‐class and K‐1 class models. A significant *p*‐value indicates model fit was significantly improved by the addition of an extra class.
- Minimum subgroup *n* (e.g., >5% of total sample).
- Model parsimony and the theoretical meaning and relevance of classes.

To control for baseline survey date and total study duration, these were regressed out of the dependent variables (loneliness at each assessment), and the residuals used in the LCGA models. After selecting the optimal LCGA model, the covariates age, sex, and country were added to the model using the 3‐step method in Mplus (Asparouhov & Muthén, 2014). The 3‐step method regresses subgroup membership on predictor variables (i.e., covariates), without allowing the inclusion of the covariates in the model to alter the latent class structure and measurement error (Asparouhov & Muthén, 2014).

Missing data in LGM and LCGA models were modelled using full information maximum likelihood estimation.

## RESULTS

### Participant characteristics

Participant characteristics for the total sample and each country are shown in Table 1. The mean age of participants was 15.47 years (SD 1.95); 64% were female. Participants in the Israel sample were younger than those in the UK and India samples, and there were more females than males in the UK sample than the India and Israel samples.

**TABLE 1:** Participant demographic characteristics and comparison between countries.

	Mean (SD)						
	Total sample	UK	Israel	India	UK versus Israel	UK versus India	India versus Israel
Age	15.47 (1.95)	15.56 (1.94)	14.87 (1.78)	15.60 (2.03)	*t*(220.62) =	*t*(292.58) =	*t*(327.91) = 3.53, *p* < 0.001
					−4.21, *p* < 0.001	0.25, *p* = 0.800	

	*N* (%)						
Sex							
Male	370 (36)	197 (28)	69 (48)	104 (54)	*Χ* ^2^(1) = 21.26, *p* < 0.001	*Χ* ^2^(1) = 46.00, *p* < 0.001	*Χ* ^2^(1) = 1.43, *p* = 0.231
Female	669 (64)	505 (72)	76 (52)	88 (46)			

### Latent growth curve model

**FIGURE 1:**
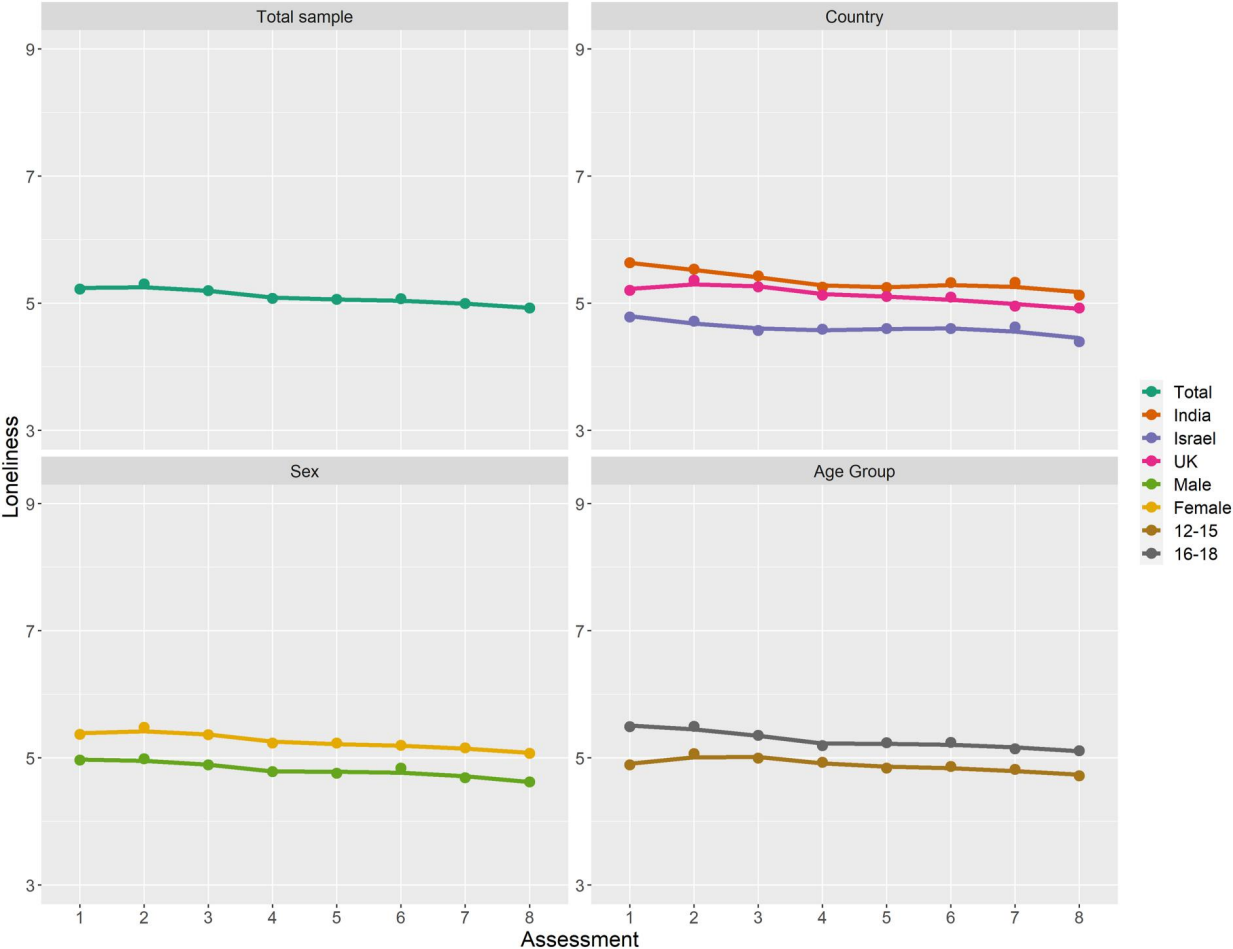
Mean trajectory of loneliness scores of participants between Assessments 1 and 8, in the total sample; UK, Israel, and India samples; males and females; 12‐15‐year‐olds and 16‐18‐year‐olds. Age was categorised into 2 age groups for illustrative purposes only; all analyses involving age were performed with age as a continuous variable. Spaghetti plots showing individual trajectories in addition to mean loneliness trajectory are shown in Figure S1.

Figure 1 shows the mean loneliness trajectory for the whole sample, and for illustrative purposes the mean loneliness trajectories for the individual countries, males and females, and younger and older adolescents are also shown. The LGM showed a significant intercept (standardised estimate = 3.54, unstandardised = 5.28, *p* < 0.001) and significant slope (standardised = −0.23, unstandardised = −0.05, *p* < 0.001), indicating that mean loneliness decreased slightly over time (*χ* ^2^(31) = 127.63, *p* < 0.001, CFI = 0.98, SRMR = 0.05). There was significant variance in the intercept (standardised = 1.00, unstandardised = 2.23, *p* < 0.001) and slope (standardised = 1.00, unstandardised = 0.04, *p* < 0.001). Because the UK sample was larger than the Israel and India samples, we performed a second LGM with country as a covariate, to check results were not driven primarily by the UK sample and ascertain whether the countries differed in terms of initial loneliness or loneliness trajectory. The overall pattern of results remained the same, and adolescents in the UK did not differ from either those in Israel or India in their loneliness trajectory, but there were some differences in initial loneliness (see Appendix S2 for full details of the results).

### Latent class growth analysis

LCGA was used to identify subgroups with different loneliness trajectories. Fit statistics for the 2 through to 6‐class models that were estimated are shown in Table 2. All LRT were significant. The model with the 5‐class solution was selected for the following reasons: (1) the improvement in fit indices between the 5‐class and 6‐class models was minimal in comparison with the improvement between the 4‐class and 5‐class models, indicating model fit was no longer substantially improving, (2) the entropy of the 5‐class model was higher than the 6‐class model, (3) the 6‐class model did not meet our minimum class size criteria (>5% of the sample), and (4) the addition of a 6th class did not add substantive meaning to the interpretation of the classes.

**TABLE 2:** Latent class growth analysis, model fit statistics.

Classes	Parameters	Convergence	Trajectories	Entropy	AIC	BIC	aBIC		Group *n* (%)	BLR	df (BLR)	*p* (BLR)
2	13	Yes	Distinct	0.870	27594.790	27659.089	27617.799	1	593 (57)	−15213.533	3	<0.001
								2	446 (43)			
3	16	Yes	Distinct	0.858	26744.066	26823.203	26772.385	1	461 (44)	−13784.395	3	<0.001
								2	383 (37)			
								3	195 (19)			
4	19	Yes	Distinct	0.821	26515.093	26609.068	26548.721	1	339 (33)	−13356.033	3	<0.001
								2	85 (8)			
								3	344 (33)			
								4	271 (26)			
5	22	Yes	Distinct	0.804	26301.859	26410.671	26340.796	1	195 (19)	−13238.547	3	<0.001
								2	347 (33)			
								3	177 (17)			
								4	85 (8)			
								5	235 (23)			
6	25	Yes	Distinct	0.794	26236.858	26360.509	26281.105	1	81 (8)	−13128.929	3	<0.001
								2	202 (19)			
								3	161 (15)			
								4	332 (32)			
								5	55 (5)			
								6	208 (20)			

*Note*: Loneliness scores are adjusted for baseline survey date and total study duration.

Abbreviations: aBIC, Sample size adjusted Bayesian information criterion; AIC, Akaike information criterion; BIC, Bayesian information criterion; BLR, Bootstrapped likelihood ratio test.

**FIGURE 2:**
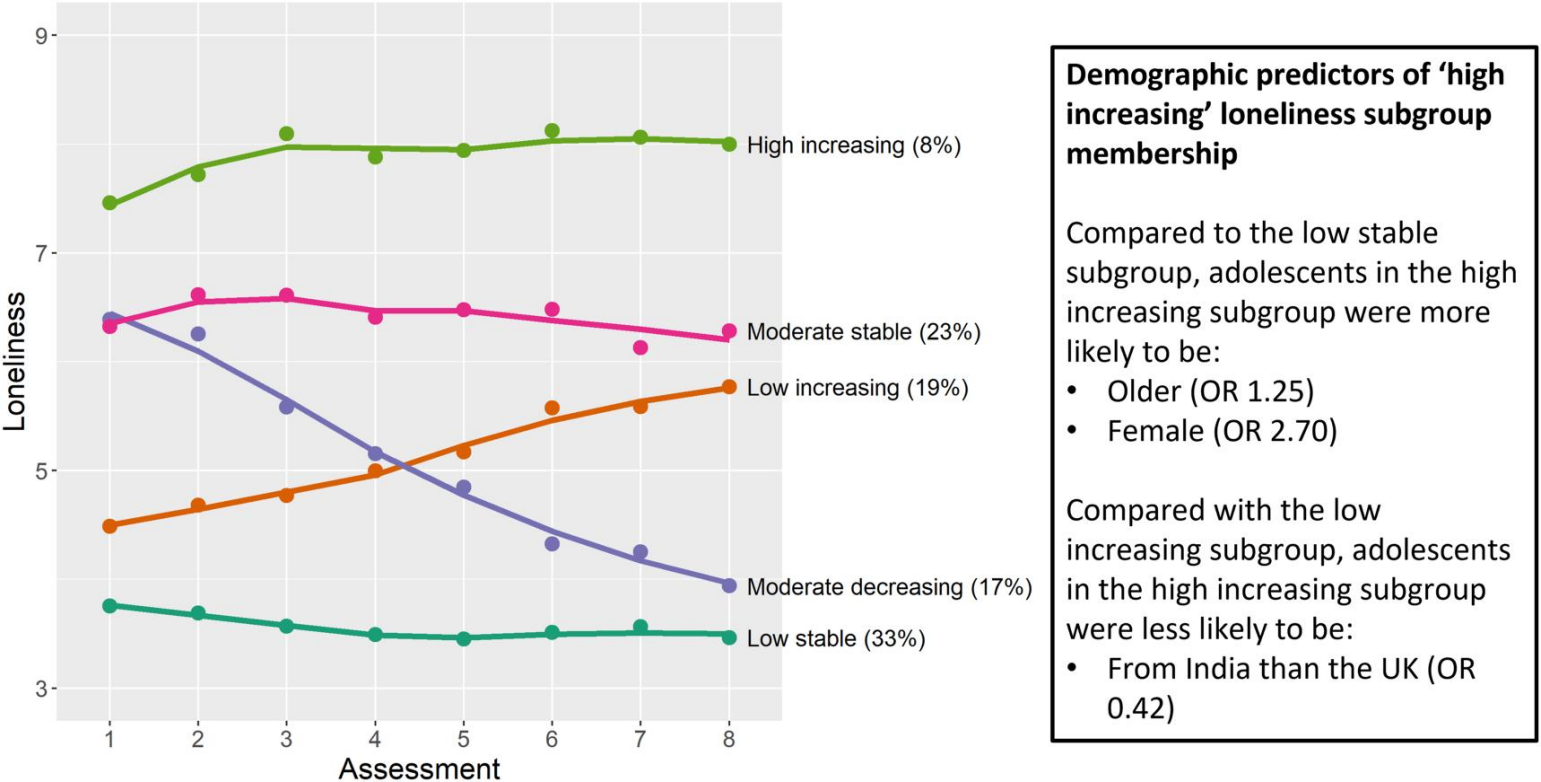
Mean trajectory of loneliness scores in different subgroups between Assessments 1 and 8. Proportion of sample in each subgroup is shown in brackets. Spaghetti plots showing individual trajectories in addition to mean loneliness trajectory of each subgroup are shown in Figure S2.

Figure 2 shows the trajectories of the 5 subgroups. The largest subgroup displayed a low stable loneliness trajectory (mean (M) intercept = −1.59, *p* < 0.001; M slope = 0.01, *p* = 0.325). There was a subgroup that displayed a relatively low initial but increasing loneliness trajectory (M intercept = −0.78, *p* < 0.001; M slope = 0.23, *p* < 0.001), a subgroup that displayed a moderate initial but decreasing loneliness trajectory (M intercept = 0.98, *p* < 0.001; M slope = −0.30, *p* < 0.001), and a subgroup that displayed a moderate stable trajectory (M intercept = 1.29, *p* < 0.001; M slope = 0.01, *p* = 0.922). The final subgroup displayed a high and increasing loneliness trajectory (M intercept = 2.37, *p* < 0.001; M slope = 0.11, *p* = 0.038).

### Predictors of loneliness subgroup membership

The results examining predictors are shown in Table 3. Participants in the high increasing loneliness subgroup were more likely to be older and female than participants in the low stable loneliness subgroup. Compared to adolescents in the UK, adolescents in India were less likely to be in the high increasing subgroup (vs. the low increasing subgroup). However, only the association between sex and subgroup membership remained significant following correction for multiple testing.

**TABLE 3:** Latent class growth analysis of loneliness predicted by demographic factors.

Variable	Loneliness subgroup comparison											
	High increasing versus Low stable loneliness			High increasing versus Low increasing loneliness			High increasing versus Moderate decreasing loneliness			High increasing versus Moderate stable loneliness		
	B (SE)	OR [95% CI]	*p*	B (SE)	OR [95% CI]	*p*	B (SE)	OR [95% CI]	*p*	B (SE)	OR [95% CI]	*p*
Age	−0.22 (0.08)	0.80 [0.69, 0.94]	0.005	−0.03 (0.09)	0.97 [0.82, 1.15]	0.722	−0.03 (0.09)	0.97 [0.82, 1.15]	0.754	−0.08 (0.09)	0.92 [0.78, 1.09]	0.347
Sex (ref male)	−0.99 (0.32)	0.37 [0.20, 0.70]	0.002*	−0.39 (0.36)	0.68 [0.33, 1.37]	0.276	−0.55 (0.35)	0.58 [0.29, 1.16]	0.123	−0.31 (0.37)	0.74 [0.36, 1.51]	0.404
Country (ref UK)												
Israel	0.26 (0.44)	1.30 [0.55, 3.04]	0.552	0.72 (0.48)	2.05 [0.80, 5.25]	0.135	−0.33 (0.56)	0.72 [0.24, 2.15]	0.558	−0.08 (0.52)	0.92 [0.33, 2.54]	0.872
India	−0.89 (0.46)	0.41 [0.17, 1.01]	0.053	0.86 (0.42)	2.37 [1.04, 5.40]	0.040	0.51 (0.42)	1.67 [0.73, 3.80]	0.223	0.52 (0.43)	1.68 [0.72, 3.92]	0.235

*Note*: Loneliness scores adjusted for baseline date and study duration.

Abbreviations: CI confidence intervals; OR odds ratio; SE standard error.

*Significant following Bonferroni correction for multiple comparisons (*p* < 0.004).

## DISCUSSION

We conducted a longitudinal study of loneliness in adolescents from the UK, Israel, and India during the COVID‐19 pandemic. The study investigated (i) changes in adolescents' loneliness over time, (ii) whether there were subgroups of adolescents with different trajectories of loneliness, and (iii) potential risk factors for experiencing persistently high loneliness in terms of age, sex, and country of residence.

Our findings indicate that although at the group level loneliness was moderate and slightly decreased with time, it was also heterogeneous. We identified five groups of adolescents with distinct loneliness trajectories: (1) low stable loneliness, (2) low increasing loneliness, (3) moderate decreasing loneliness, (4) moderate stable loneliness, and (5) high increasing loneliness. Although a third of the sample were in the low stable loneliness group, it is concerning that around half of adolescents showed either an increasing or consistently moderate level of loneliness, and 8% showed high and increasing loneliness. A longitudinal study of loneliness in UK adults at the start of the pandemic identified 4 groups with different loneliness trajectories, one of which also showed persistently high loneliness and represented around 14% of the total sample (Bu et al., 2020). Although we are unable to estimate prevalence as our samples are not representative of their respective countries, our findings are somewhat similar to pre‐pandemic prevalence rates of loneliness in young people in the UK. Research by the ONS found that around a third of 10‐15‐year‐olds reported almost never feeling lonely, whereas around 14% reported experiencing frequent loneliness (Office for National Statistics, 2018a). As there are no comparable national statistics in India and Israel, it is difficult to benchmark our findings against prevalence rates in these countries.

In our study, the most robust finding was that females were at higher risk than males of experiencing persistently high loneliness. Pre‐pandemic research investigating the male to female differences in loneliness in this age group have produced mixed findings. Some cross‐sectional studies report higher rates of loneliness in females (Moksnes et al., 2022; Yang et al., 2020), but previously no association between sex and loneliness trajectory in youth has been found in longitudinal studies (Schinka et al., 2013; Vanhalst et al., 2013). These were, however, developmental studies examining changes in loneliness over a period of years, rather than months as was the case in the current study. Our finding is in agreement with Bu et al. (2020), who also found that adult females were more likely than males to experience higher loneliness over the early stages of the pandemic. It is possible that the lockdowns implemented in response to the pandemic had a greater effect on females' existing social behaviours and reliance on social support than males', resulting in higher loneliness.

Older adolescents were at higher risk than younger adolescents of experiencing persistently high loneliness. Although this finding did not survive correction for multiple testing and should therefore be interpreted with caution, it is in line with previous research in adult samples indicating that late adolescence and early adulthood are key periods for experiencing heightened loneliness (Barreto et al., 2021; Victor & Yang, 2012). However, in contrast with our findings, the ONS reported that loneliness may be higher in very early adolescence (10–12 years), than mid‐adolescence (13–15 years) (Office for National Statistics, 2018a). There are many differences between the ONS study and the current study that could explain this discrepancy: the ONS study is cross‐sectional, only includes young people from the UK, has a lower age range, and was conducted before the pandemic. For example, it may be that the transition from primary to secondary school could result in higher loneliness at this age (11 years), which would not have been captured by our study. It is also possible that the restrictions to (in‐person) social interactions due to the pandemic affected older adolescents' socialising more than younger adolescents', resulting in higher loneliness in this age group.

Finally, we found that adolescents from the UK were at higher risk of experiencing persistently high loneliness than adolescents from India. Previous cross‐country comparisons of loneliness in youth are very limited: to our knowledge no other longitudinal study has investigated this, and cross‐sectional studies have reported no difference in mean loneliness between countries (Chen et al., 2004; Maes et al., 2016). In the current study, differences between the countries' lockdowns and restrictions to ‘in‐person’ socialising due to the pandemic may have affected cross‐country comparisons. As this finding did not survive correction for multiple testing, further exploration is warranted before firm conclusions are drawn on this cross‐country difference.

The current study has several strengths: it is the first to explore longitudinal changes in adolescent loneliness during the COVID‐19 pandemic, and it expands this beyond Western countries using a relatively large sample which covers a period of development spanning from early to late adolescence. Unlike previous longitudinal studies investigating loneliness over a period of years, we have captured more rapid changes in loneliness in response to a potent stressor (the COVID‐19 pandemic) over a shorter period of time. The study also has some limitations that should be noted. Loneliness was measured using a version of the 3‐item UCLA loneliness scale (Hughes et al., 2004), which was adapted and tested qualitatively in a collaboration between the ONS and The Children's Society and is recommended for use in those aged 10–15 years (Office for National Statistics, 2018b, 2018c). This ensured our measure of loneliness was age‐appropriate and quick to administer given the extended questionnaire battery. However, it should be noted that current measures of youth loneliness have been criticised for often i) not including sufficient items to reflect the multidimensional experience of loneliness and reduce potential stigma (What Works Centre for Wellbeing, 2019), ii) not having been developed with input from young people, thereby potentially failing to capture their authentic loneliness experiences (Cole et al., 2021), iii) have undergone limited psychometric testing (Cole et al., 2021), and iv) are unable to screen for those at risk for more severe forms of loneliness (Cole et al., 2021). The sample was not random and not selected to be nationally representative of the respective countries; this means that we weren't able to estimate prevalence and there are concerns about generalisability to other peers in the same countries. Different recruitment methods were used in each country, and the countries also differed in terms of COVID‐related restrictions at the time of the study, therefore country‐based comparisons should be interpreted with caution. Furthermore, response bias may have been introduced by including only those adolescents who were able to access the surveys, those who were particularly motivated to take part, and those were motivated to take part for reimbursement and may therefore have responded more carelessly. The UK and Israeli adolescents were reimbursed for taking part, but adolescents in India were not, which may also confound country‐based differences. As this was a multi‐wave study, it is important to consider the potential impact of attrition bias on our findings. For the purpose of statistical rigour, we only included participants who had data for at least 5 time points. However, we noted that males and participants in the UK and Israel were more likely to be excluded due to having insufficient data points. This should therefore be considered when interpreting the current findings. In addition to sex, age, and country, there are other potential moderators of loneliness trajectories that we did not measure and were therefore unable to include in our analyses, including gender (McDanal et al., 2023), mental and physical health (Hutten et al., 2021; Qualter et al., 2013; Schinka et al., 2013; Vanhalst et al., 2013), socioeconomic status (Moksnes et al., 2022; Qualter et al., 2021; Schinka et al., 2013), and ethnicity (Yang et al., 2020). The surveys were conducted online, raising questions about measurement error. Finally, we did not have data from before the pandemic, and were therefore unable to make comparisons of pre and during pandemic experiences of loneliness.

## CONCLUSION

Our findings suggest that adolescents' loneliness over time during the COVID‐19 pandemic was relatively heterogeneous, and specifically a significant proportion of adolescents were experiencing persistently high or increasing loneliness. Among these, females and older adolescents were at higher risk of experiencing high and persistent levels of loneliness and may therefore benefit most from guidance and intervention. Future research exploring trajectories of adolescent loneliness in a wider range of countries, and comparing pre‐ and post‐pandemic loneliness, would be particularly valuable.

## AUTHOR CONTRIBUTIONS

**Laura Riddleston**: Conceptualization; data curation; formal analysis; investigation; methodology; project administration; visualization; writing – original draft; writing – review & editing. **Meenakshi Shukla**: Data curation; investigation; project administration; writing – review & editing. **Iris Lavi**: Data curation; funding acquisition; investigation; project administration; writing – review & editing. **Eloise Saglio**: Conceptualization; formal analysis; writing – original draft. **Delia Fuhrmann**: Conceptualization; formal analysis; methodology; supervision; writing – review & editing. **Rakesh Pandy**: Investigation; writing – review & editing. **Tushar Singh**: Investigation; writing – review & editing. **Pamela Qualter**: Conceptualization; formal analysis; methodology; supervision; writing – review & editing. **Jennifer Lau**: Conceptualization; formal analysis; funding acquisition; methodology; supervision; writing – original draft; writing – review & editing.

## CONFLICT OF INTEREST STATEMENT

The authors have declared that they have no competing or potential conflicts of interest.

## ETHICS STATEMENT

The UK study received ethical approval from the Psychiatry, Nursing and Midwifery Research Ethics Committee at Kings College London (Ref: HR‐19/20–18250), the Israel study from the Ethics Committee for Human Experiments, University of Haifa (Ref: 368/20) and from the Psychology Ethics Committee, University of Bath (Ref: 4688 20–05469), and the India study from the Institutional Ethics Committee, Institute of Medical Sciences, Banaras Hindu University, India (Ref: Dean/2020/EC/1975).

## Supporting information

Supporting Information S1

## Data Availability

The data that support the findings of this study are available from the corresponding author upon reasonable request.
