# Supplementary material for: Identifying characteristics of adolescents with persistent loneliness during COVID‐19: A multi‐country eight‐wave longitudinal study
Source: JCPP Adv. 2023 Nov 8;4(1):e12206. doi: 10.1002/jcv2.12206 (PMC10933679; doi:10.1002/jcv2.12206)
Supplement: Supplementary file 1 — Supporting Information S1 [file JCV2-4-e12206-s001.docx]

**Supporting Information**

|  | **UK** |  | **India** |  | **Israel** |  |
| --- | --- | --- | --- | --- | --- | --- |
| **Assessment** | **Earliest-Latest date** | **Range (days)** | **Earliest-Latest date** | **Range (days)** | **Earliest-Latest date** | **Range (days)** |
| 1 | 12/05/20-24/11/20 | 196 | 05/06/20-12/07/20 | 37 | 17/05/20-16/06/20 | 30 |
| 2 | 14/06/20-02/12/21 | 171 | 20/06/20-04/08/20 | 45 | 31/05/20-12/07/20 | 42 |
| 3 | 28/06/20-09/01/21 | 195 | 05/07/20-18/09/20 | 75 | 14/06/20-30/07/20 | 46 |
| 4 | 11/07/20-23/01/21 | 196 | 20/07/20-04/10/20 | 76 | 28/06/20-10/08/20 | 43 |
| 5 | 25/07/20-10/02/21 | 200 | 04/08/20-19/10/20 | 76 | 12/07/20-11/09/20 | 61 |
| 6 | 08/08/20-21/03/21 | 225 | 19/08/20-18/10/20 | 60 | 26/07/20-10/09/20 | 46 |
| 7 | 22/08/20-12/03/21 | 202 | 04/09/20-23/10/20 | 49 | 09/08/20-13/09/20 | 35 |
| 8 | 05/09/20-16/04/21 | 223 | 18/09/20-24/10/20 | 36 | 23/08/20-11/09/20 | 19 |

**TableS1.** Survey assessment dates.

|  | **UK** | **India** | **Israel** |
| --- | --- | --- | --- |
| **Assessment** | **Mean time between surveys (Days)** | **Mean time between surveys (Days)** | **Mean time between surveys (Days)** |
| 1-2 | 18 | 18 | 18 |
| 2-3 | 16 | 16 | 16 |
| 3-4 | 15 | 15 | 15 |
| 4-5 | 18 | 16 | 19 |
| 5-6 | 17 | 16 | 17 |
| 6-7 | 16 | 16 | 16 |
| 7-8 | 16 | 15 | 18 |

**TableS2.** Length of time between assessments.

| **Assessment** | **Total Sample** | **UK** | **Israel** | **India** |
| --- | --- | --- | --- | --- |
| **1** | 1039 | 702 | 145 | 192 |
| **2** | 1039 | 702 | 145 | 192 |
| **3** | 1039 | 702 | 145 | 192 |
| **4** | 1039 | 702 | 145 | 192 |
| **5** | 1039 | 702 | 145 | 192 |
| **6** | 916 | 619 | 120 | 177 |
| **7** | 799 | 552 | 86 | 161 |
| **8** | 659 | 487 | 48 | 124 |

**TableS3.** Sample size (*n*) at each assessment, for the total sample and individual countries.

**Appendix S1.** Adapted 3-item UCLA loneliness scale.

Adapted version of the 3-item UCLA loneliness scale [[1]](https://sciwheel.com/work/citation?ids=1033864&pre=&suf=&sa=0&dbf=0) recommended for use with children and young people [[2]](https://sciwheel.com/work/citation?ids=11248963&pre=&suf=&sa=0&dbf=0):

In the last two weeks, how often have you…

1. Felt that you have no one to talk to?
2. Felt left out?
3. Felt alone?

Response options ‘Hardly ever/Never’, ‘Some of the time’, and ‘Often’.

**
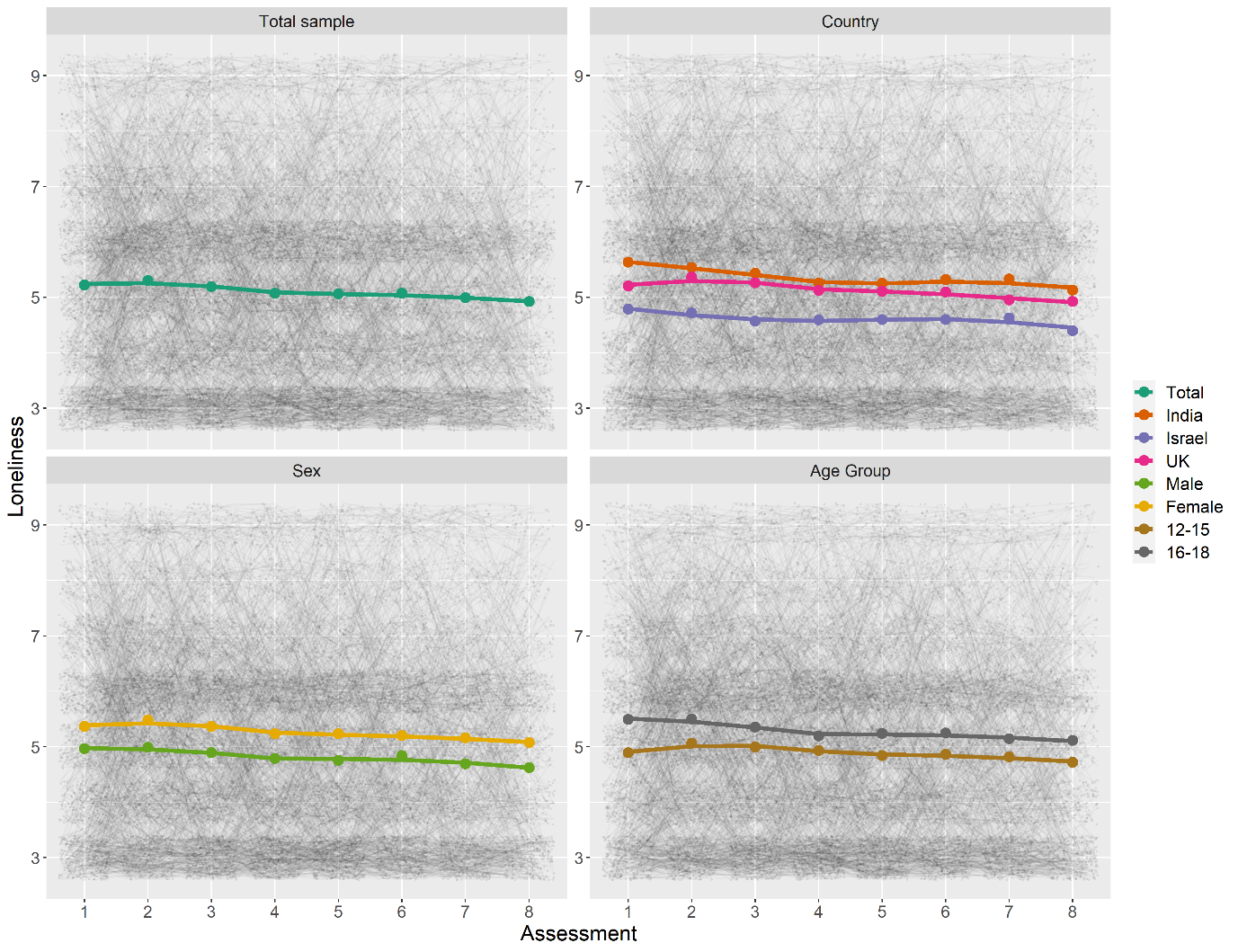
 FigureS1.** Mean trajectory and spaghetti plots of loneliness scores of participants between Assessments 1 and 8, in the total sample; UK, Israel, and India samples; Males and females; 12-15-year-olds and 16-18-year-olds. Note: Age was categorised into 2 age groups for illustration purposes only; all analyses involving age were performed with age as a continuous variable.

**Appendix S2.** Latent growth curve model (LGM) results with country as a covariate.

The LGM showed a significant intercept (standardised estimate=3.56, unstandardised=5.32, *p*<.001) and a significant slope (standardised=-0.23, unstandardised=-0.05, *p*<.001), indicating that mean loneliness decreased slightly over time (χ^2^(43)=137.90, p<.001, CFI=0.98, SRMR=0.04). The results also indicated significant variance in the intercept (standardised=0.97, unstandardised=2.16, *p*<.001) and slope (standardised=1.00, unstandardised=0.04, *p*<.001). Compared with adolescents in the UK, adolescents in Israel had lower initial loneliness (β=-0.41, p<.001), and adolescents in India had slightly higher initial loneliness (β=0.17, p=0.044). Adolescents in the UK did not differ from either those in Israel or in India in their trajectory of loneliness over time (all p>.05).


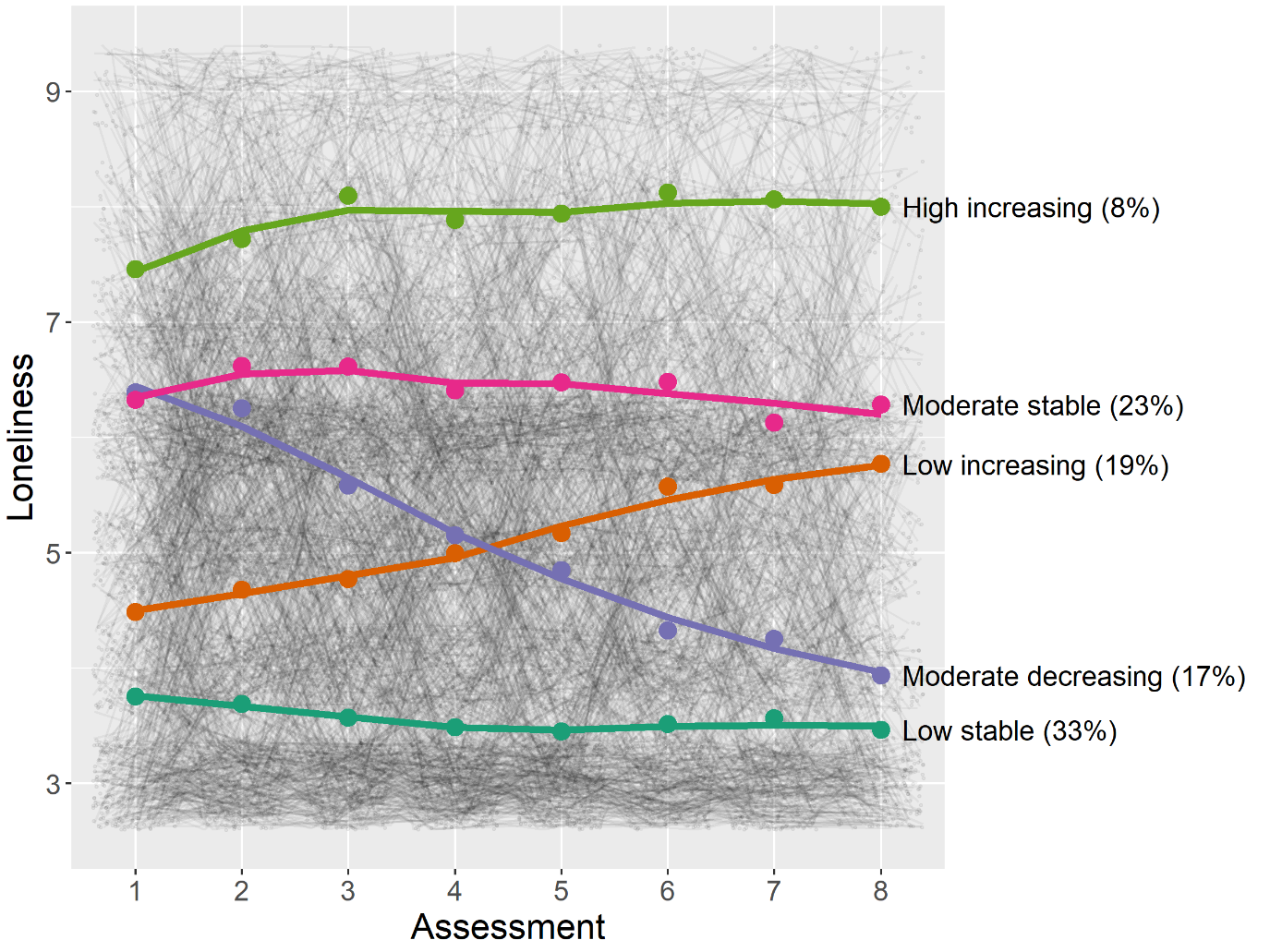


**FigureS2.** Mean trajectory and spaghetti plots of loneliness scores in different subgroups between Assessments 1 and 8. Proportion of sample in each subgroup shown in brackets.

**References**

[[1]     Hughes ME, Waite LJ, Hawkley LC, et al. A Short Scale for Measuring Loneliness in Large Surveys: Results From Two Population-Based Studies. Res Aging 2004;26:655–72.](https://sciwheel.com/work/bibliography/1033864)

[[2]     Office for National Statistics. Measuring loneliness: guidance for use of thenational indicators on surveys. Available at: https://www.ons.gov.uk/peoplepopulationandcommunity/wellbeing/methodologies/measuringlonelinessguidanceforuseofthenationalindicatorsonsurveys. AccessedJune 22, 2021.](https://sciwheel.com/work/bibliography/11248963)
